# Small RNA Mobility: Spread of RNA Silencing Effectors and its Effect on Developmental Processes and Stress Adaptation in Plants

**DOI:** 10.3390/ijms20174306

**Published:** 2019-09-03

**Authors:** Chiara Pagliarani, Giorgio Gambino

**Affiliations:** Institute for Sustainable Plant Protection, National Research Council (IPSP-CNR), 10135 Torino, Italy

**Keywords:** small RNAs, systemic silencing, grafting, environmental adaptation, epigenetic modifications, stress memory

## Abstract

Plants are exposed every day to multiple environmental cues, and tight transcriptome reprogramming is necessary to control the balance between responses to stress and processes of plant growth. In this context, the silencing phenomena mediated by small RNAs can drive transcriptional and epigenetic regulatory modifications, in turn shaping plant development and adaptation to the surrounding environment. Mounting experimental evidence has recently pointed to small noncoding RNAs as fundamental players in molecular signalling cascades activated upon exposure to abiotic and biotic stresses. Although, in the last decade, studies on stress responsive small RNAs increased significantly in many plant species, the physiological responses triggered by these molecules in the presence of environmental stresses need to be further explored. It is noteworthy that small RNAs can move either cell-to-cell or systemically, thus acting as mobile silencing effectors within the plant. This aspect has great importance when physiological changes, as well as epigenetic regulatory marks, are inspected in light of plant environmental adaptation. In this review, we provide an overview of the categories of mobile small RNAs in plants, particularly focusing on the biological implications of non-cell autonomous RNA silencing in the stress adaptive response and epigenetic modifications.

## 1. Introduction

The way a plant responds to environmental stimuli during its lifetime is guided by an intricate and coordinated array of physiological and biochemical events [1,2]. The mechanisms of gene regulation represent one of the most important and studied phenomena. Tight transcriptome reprogramming is indeed necessary to take control of the balance between responses to stressful conditions and plant growth [3,4,5]. In recent years, mounting experimental evidence has demonstrated that silencing processes triggered by small noncoding RNAs (sncRNAs) are the basis for those transcriptional (i.e., transcriptional gene silencing, TGS) and post-transcriptional (i.e., post-transcriptional gene silencing, PTGS) regulatory pathways which fine tune different facets of the everyday plant life, including development and physiological adaptation to the surrounding environment [6].

Based on their biogenesis, sncRNAs are classified into two main classes: short-interfering RNAs (siRNAs) and microRNAs (miRNAs). Each exert specific functions in gene regulation that often overlap with hormonal signalling cascades.

miRNAs are small RNA sequences 20–22 nt in length that originate in the cell nucleus through DICER like 1 (DCL1)-induced cleavage of single-stranded stem-loop precursors transcribed by RNA polymerase II (Pol II) from endogenous *MIR* genes [7,8]. Once delivered to the cytoplasm, mature miRNAs bind to specific AGO proteins to form the AGO-associated RNA-induced silencing complex (RISC) and act as a guide to mediate either cleavage or translational inhibition of complementary target mRNAs [9,10]. Although most miRNAs are primarily known as post-transcriptional silencing effectors, a few of them, with longer sequences about 23–25 nt in length, have been shown to enter the RNA-directed DNA methylation (RdDM) pathway. Those miRNAs induce chromatin modifications at target loci via triggering the production of specific siRNAs, as attested by experiments carried out in *Arabidopsis*, rice, and maize [11,12,13,14].

miRNAs have long been established as key regulators of developmental processes in plants [15,16]. Particularly in the last decade, their master role in mediating signalling cascades in response to abiotic and biotic stresses has clearly emerged, as highlighted in many excellent review papers [4,6,17,18,19]. Progress in high-throughput sequencing technology and the availability of dedicated bioinformatic tools have progressively allowed the identification of many conserved and novel miRNA sequences targeting transcripts that are typically modulated by abiotic and biotic cues, as evidenced by an increasing number of studies conducted in model herbaceous species [20,21,22] and several important crops [23,24,25,26,27], including fruit trees [28,29,30,31,32].

Unlike miRNAs, siRNAs are synthesised from long double stranded RNA precursors through the action of different DCL enzymes (DCL2, DCL3 or DCL4, depending on the siRNA class), and they are primarily involved in TGS, therefore playing a pivotal role in the maintenance of genome stability.

One additional important function of siRNA generation in plants is in antiviral defence mechanisms [33]. The endogenous siRNAs thus far characterised in plants comprise: the 23 to 24 nt long heterochromatic siRNAs (hc-siRNAs), the 21 to 22 nt long siRNAs, which, in turn, include trans-acting siRNAs (ta-siRNAs), phased siRNAs (pha-siRNAs) and epigenetically-activated siRNAs (ea-siRNAs), and the natural antisense siRNAs (nat-siRNAs) [34]. Heterochromatic siRNAs are associated with transcriptional silencing at the transposon and pericentromeric repeat levels through activation of RdDM [35], and they arise from intergenic and repetitive genomic regions transcribed by Pol IV. The transcribed precursor is then processed by RDR2 into dsRNA, which is diced by DCL3 to form 23 to 24-nt long hc-siRNAs. Unlike hc-siRNAs, the biogenesis of 21 to 22 nt siRNAs involves a different set of enzymes. It results from the action of DCL4 and DCL2, following Pol II transcription of the primary transcript and dsRNA synthesis by RDR6 [34]. Another distinct feature of this pathway is that the generation of ta-siRNA and pha-siRNA precursors requires the action of one or more upstream miRNAs targeting transcripts at the *TAS* or *PHAS* loci, respectively [36,37].

The RNA silencing response can be amplified when siRNAs derived from the target sequence (primary siRNAs) initiate the production of a second wave of siRNAs from the regions located outside their primary target sites, which are called secondary or transitive siRNAs [38]. The spreading of RNA silencing beyond the initial target sites is thus referred to as transitivity, and it depends on both (i) the sncRNA trigger and (ii) the mRNA target. (i) Primary sncRNAs that are 22 nt in length (miRNAs or siRNAs [39]) or containing an asymmetric bulge [40] bind to AGO1 and recruit RDR6 to their mRNA target site, leading to secondary siRNA formation. Moreover, (ii) mRNAs transcribed from intronless genes are more susceptible to RDR6 processing and secondary siRNA formation than mRNAs originating from intron-containing genes [41].

Besides DNA methylation, secondary siRNAs, such as ta-siRNAs, can trigger the degradation of target mRNAs and cause translational repression, just as miRNAs can [35]. Furthermore, Brodersen et al. previously provided experimental evidence supporting that siRNA populations may induce translational inhibition, in addition to slicing target mRNAs [42].

The biogenesis and biological role of nat-siRNAs is still far from being elucidated; however, available information indicates that it does not depend on RDR activity for the generation of dsRNA precursors. Indeed, nat-siRNA precursors are derived by the hybridisation of two complementary RNAs that are transcribed separately, either from opposite strands of two overlapping genes (*cis*-nat-siRNAs) or from non-overlapping genes sharing mRNA complementarity (*trans*-nat-siRNAs) [34].

Although, in recent years, there was an explosion of research highlighting the biological function of plant small RNAs in developmental and stress responses [4,16,18], many features of the RNA signalling pathways are still poorly understood. The transport of these molecules within the plant and their consequent effects, including the transgenerational inheritance of epigenetic marks underlying stress memory phenomena, require further investigations to be deciphered completely [43]. Finally, another noteworthy aspect pertains to the flexible regulation of small RNA biogenesis in response to specific stress signals [44].

In this review, we provide an overview of the main categories of mobile small RNAs exerting their activities in plants. Then, we discuss the biological implications of the non-cell autonomous spread of RNA silencing effectors (in particular, miRNAs) in light of plant development and stress adaptive strategies.

## 2. A Focus on Non-Cell Autonomous Signalling Phenomena Mediated by Small RNAs

**Figure 1:**
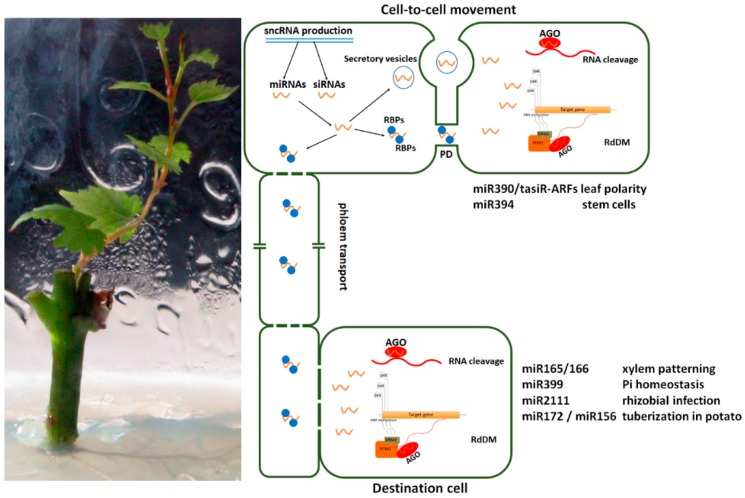
Schematic overview of non-cell autonomous RNA delivery in plant tissues and potential biological activity in recipient cells. siRNAs and miRNAs can act as mobile signals moving cell-to-cell through plasmodesmata (PD), which establish cytosolic continuity between adjacent cells, and through vesicles resembling exosomes (hypothesis). Long distance trafficking of siRNAs and miRNAs, putatively complexed with protein associated to RNA (RBPs), takes place through the phloem stream, preferentially following the source-to-sink gradient. In recipient cells, the 21 to 22-nt long small RNAs incorporated into the ARGONAUTE 1 (AGO1) effector protein induce cleavage of complementary target mRNAs. The 23 to 24 nt long siRNAs loaded onto AGO 4 and/or 6 guide de novo DNA methylation (belonging to RNA directed-DNA methylation (RdDM) maintenance pathways) in a sequence-specific manner. The image on the left shows a micro-grafted grapevine plantlet grown under in vitro conditions.

The first evidence revealing the spread of RNA silencing in plants was derived from the investigations of viral RNAs that can move cell-to-cell and systemically through the vasculature [45]. However, over the last twenty years, it has also become clear that endogenous sncRNAs and proteins can be unloaded into proximal and distant cells. Non-cell autonomous transport of RNAs within the whole plant involves both local and systemic movement. The local spread, characterised by a transport between adjacent cells, is coupled both in the source tissue of the signal and in the destination tissue, with a systemic movement involving the vascular system (Figure 1).

Short-distance delivery of RNAs occurs in a cell-to-cell manner and, essentially, via plasmodesmata (PD), which establish cytosolic continuity between adjacent cells (i.e., symplastic movement) (Figure 1). In addition to water, and depending on their size and recipient tissues, diverse types of small molecules can be transported through PD pores, such as ions, metabolites, hormones, proteins, mRNAs and sncRNAs. Therefore, the PD act as dynamic channels that can undergo structural and functional changes also induced by specific plant developmental stages or environmental stressors. This theory is supported by the fact that many sncRNAs involved in local movement, particularly miRNAs, are key regulators of specific developmental processes (Table 1). Notably, recent investigations based on in vivo visualization assays experimentally confirmed the cell-to-cell movement of RNAs through PD [46]. Further experiments elucidated that the PD size is tightly regulated via reversible callose deposition, provided by the synergistic action of callose synthases and beta-glucanases associated with PD [47]. Much of the knowledge concerning PD-mediated trafficking came from the study of the intercellular and systemic transport of pathogens, such as plant viruses and viroids. Viruses typically encode movement proteins (MP) that interact with nucleic acids, the microtubule cytoskeleton and other cellular components in order to modify the PD pore size and to allow the release of viral particles into neighbouring cells [48]. Additionally, since viroids must use only host proteins for their translocation [49], they represent an ideal system to gain insights into the intercellular transport of endogenous RNA molecules.

In addition to PD, vesicles have been proposed as an alternative path facilitating the cell-to-cell transport of RNAs (Figure 1). Plant vesicles resemble the exosomes observed in animal tissues, which consist of membrane-bound vesicles derived from multivesicular bodies (MVBs) that are released by exocytosis after fusion with the plasma membrane [67]. Some experimental proof is available in support of the hypothesis that exosome-like vesicles also exist in plants [68,69]. In particular, the enhanced production of vesicles in response to biotic stress has been reported in *Arabidopsis* [70]. Although it has not yet been proven that vesicles are able to incorporate RNA molecules, proteomic analyses carried out on them revealed an enrichment in proteins involved in stress responses, coupled with an overlap between the vesicle and PD proteomes. These findings identified the PD as a hotspot for the transfer of extracellular vesicles in specific environmental conditions [71].

The long-distance trafficking of molecules typically occurs through the plant vascular system. While water and minerals are commonly transported from root-to-shoot through the xylem vessels, photosynthates and macromolecules move along the phloem stream following the source (leaf, shoot)-to-sink (root, fruit) direction. Since RNA molecules and ribonucleoprotein complexes were already isolated from phloem exudate [72,73], and previous work supported the absence of RNase activity in phloem sap samples, the phloem vessels have been established as an ideal route for RNA translocation [74] (Figure 1). Nevertheless, recent experiments carried out using exogenously applied RNAs supported the existence of an alternative transportation route. Indeed, Dalakouras et al. [75] observed that trunk injected and/or petiole-absorbed hairpin RNAs and/or small RNAs are exclusively transported through the xylem conduits in both herbaceous and woody species.

### 2.1. Mechanisms Underlying Small RNA Mobility

Systemic spreading of RNA silencing effectors was originally demonstrated in grafting experiments coupled with the use of transgenic plants [76]. Agroinfiltration of GFP transgenes was also successfully applied, proving the existence of mobile signals associated with transcriptional and post-transcriptional gene regulation [77].

The first indications that sncRNAs act as mobile signals for the establishment of silencing phenomena were reported by Smith et al. and by Molnar et al. [64,78]. Additionally, several sequences of sncRNAs were identified in the phloem sap samples collected from several plants [54,55,58,79,80]. Although miRNAs appear relatively less mobile than siRNAs, transport was documented for a few, such as miR399, miR169, miR156, miR172 and miR2111 (Table 1).

It must be emphasized that the biology underlying small RNA mobility has yet to be explored in depth. One general assumption is that 21-nt sncRNAs mediate RNA silencing over short distances, while 24-nt sncRNAs are the effectors of silencing over long distances. Nevertheless, it was shown that both molecules are capable of moving cell-to-cell or systemically; therefore, it is conceivable that biological triggers, other than sRNA size, control which of the two siRNA classes is effective. An early breakthrough into this scenario was advanced by de Felippes et al. in a study involving the use of artificial ta-siRNAs and miRNAs sharing the same sequences [81]. These authors analysed the mobility of artificial RNA molecules in *Arabidopsis* plants, proving that ta-siRNA-based silencing can spread into a much broader number of cells than miRNA-based silencing. This suggests that the specific biogenetic pathway of sncRNAs can affect their transport. However, it must be noted that artificial sncRNAs were used in this study, thus more investigations are undoubtedly required to confirm these findings, especially using endogenous classes of siRNAs and miRNAs. In addition, the nature of mobile signalling effectors should be investigated more in depth. For instance, Brosnan et al. [82] showed that, in *dcl1–8* and *dcl2 dcl3 dcl4* knockout mutants of *Arabidopsis*, miRNA and siRNA biogenesis were repressed, respectively. However, when WT *Arabidopsis* shoots were grafted onto those genotypes, transmission of the long-distance silencing from the rootstock was not compromised. Therefore, they concluded that the activities of the four *Arabidopsis* DCL enzymes were not necessary to generate systemic RNA signals, and that long sRNA precursors could act as mobile signals [82].

A subject that also needs further exploration is whether sncRNAs are transferred in complexes with proteins (i.e., DICER-like, AGO) or via transporter activity. For instance, the delivery of endogenous sncRNAs coupled with RNA-binding proteins was first hypothesised by analysing phloem exudates collected from pumpkin and *Brassica napus* plants [58,83]. Recently, Calderwood et al. suggested the existence of a passive, non-selective pathway which depends on mRNA abundance and transcript length and/or gradient (ie., mRNAs of smaller size had higher stability, a longer life and were resultingly more mobile) [84]. In parallel, other experimental results indicated an active mobility of these molecules through selective pathways. In this case, it was proposed that the recognition of specific sequence motifs, such as tRNA-like structures (TLS), can trigger mRNA movement. This implies that proteins associating with RNAs (RBPs) interact with mobile RNAs [85]. Accordingly, a large number of mobile transcripts contain TLS motifs in the coding sequence or UTRs [86]. In addition, RBPs putatively involved in RNA stability and in the loading of RNAs and sncRNAs were identified in the phloem of several plant species [46]. In phloem sap samples taken from pumpkin (*Cucurbita maxima*), the PHLOEM SMALL RNA-BINDING PROTEIN1 (PSRP1) selectively bound to 25-nt single-stranded RNAs, mediating their transport in a cell-to-cell manner [83]. Despite these research efforts, information on the nature of RNA transport mechanisms is still elusive. Indeed, it is still not well determined how RNA movement occurs mechanistically, and significant gaps remain in our understanding of the single translocation steps associated with the systemic spread of RNAs [87].

Other open questions involve the directionality of silencing transport. It was advanced that sncRNAs generated from transgenes have the capacity to move root-to-shoot via repeating cell-to-cell mechanisms [88], whereas sncRNAs produced from endogenous loci are transported via the phloem conduits following the sugar gradient (source-to-sink).

Furthermore, although the bidirectionality of RNA trafficking was more predominantly supported for siRNAs than for miRNAs, recent works suggest that miRNAs can also move shoot-to-root and vice-versa. Pan et al. demonstrated the stress-inducible root-to-shoot transport of miR172a molecules in grafting experiments, in which wild-type soybean shoots were grafted onto overexpressing hairy roots (172-OHR) or control hairy roots (CHR) stocks [56]. The expression of miR172a was significantly higher in the leaves from heterografts than in those from control plants. Accordingly, transcription rates of the *salt suppressed AP2 domain-containing 1* (*SSAC1*) gene, the target of miR172a, decreased in the same samples, following a divergent pathway.

### 2.2. Effects of Small RNA Trafficking in Plant Development and Stress Response

The transport of RNAs within plants represents a clever evolutionary strategy to control and shape a large series of physiological processes linked to development and stress responses (Table 1). It is well known that sncRNAs act as silencing effectors to withstand pathogen spread [89]. In addition, some siRNAs can be transferred from plants to pathogens to inhibit the expression of virulence genes, a phenomenon resulting in cross-kingdom gene silencing mechanisms [19,90,91]. In parallel, regarding miRNA translocation, Carlsbecker et al. reported one of the first examples of cell-to-cell movement of miRNAs, describing the effects of miR165/166 trafficking in the roots of *Arabidopsis* [51]. miR165/166 regulates a broad class of III homeodomain leucine zipper (HD-ZIPIII) transcription factors associated with embryo and meristem development, as well as with organ polarity and vascular development (Baucher et al. [92] and references therein). Interestingly, the authors highlighted the existence of a bidirectional cell signalling pathway involving miRNA165/166 and two transcription factors (i.e., Short root and Scarecrow SHR/SCR) controlling xylem patterning. SHR/SCR produced in the vascular cylinder move into the root endodermis to activate *MIR165a* and *MIR166b* transcription. The mature miR165/166 sequences generated from the endodermis are then unloaded into the vascular cylinder to target the HD-ZIP III transcription factors, thereby determining the differentiation of xylem cell types in a dosage-dependent manner [51]. Miyashima et al. later emphasised these aspects [52]. They showed that endodermis-derived miR165 controls multiple differentiation status in the root by acting in a dose-dependent manner to form a graded distribution of *HD-ZIP III* transcripts across the entire stele (Table 1).

Other confirmations in miRNA transport were achieved by studying the miR390 responsible with AGO7 for the production of *TAS3*-derived secondary siRNAs (tasiR-ARFs) [93]. Chitwood et al. documented the intercellular movement of endogenous tasiR-ARFs from their source of biogenesis, (on the upper (adaxial) side of leaves) to the lower (abaxial) leaf side [50]. Based on these findings, the authors concluded that the cell-to-cell mobilisation of miR390 in leaf cells served as the upper trigger for the graded accumulation of ARF3, following the production of tasiR-ARF from the miR390-mediated cleavage of *TAS3* loci. Besides miR390, miR394 was also reported to serve as a mobile signal regulating the patterning of stem cells. miR394 sequences generated at the protoderm level confer stem cell competence to the distal meristem by repressing transcripts encoding the F box Leaf Curling Responsiveness factor [53]. In more detail, the researchers found that, while *MIR394* transcription was restricted only at the L1 cell layer, the accumulation of mature miR394 followed a decreasing gradient across the meristem cell layers, causing the repression of F box targets in the sub-epidermal cells of the shoot meristem. Interestingly, miR394 seems unable to travel over large distances, as only three cell layers are affected by its movement. Most likely, this depends on the restricted number of cells involved in stem cell competence.

The phloem sap of oilseed rape (*Brassica napus*) was shown to contain many sncRNAs. Interestingly, the levels of the miRNAs known to respond to nutrient deprivation, such as miR395 (sulphate) [94], miR398 (copper) [95] and miR399 (phosphate) [59], increased in the phloem conduits during conditions of plant growth under starvation [58] (Table 1). Systemic signalling of miR399, which is involved in the control of inorganic phosphate (P_i_) homeostasis by tuning the expression of the *ubiquitin conjugating E2 enzyme* (*PHO2*) encoding gene, was elucidated using transgenic grafted plants [60]. Notably, miR399* is also accumulated under P_i_ deficiency and transferred across the graft junction, therefore supporting its potential biological role in P_i_ homeostasis [96]. In another study, also conducted on sap exudates from oilseed rape plants, Pant et al. identified miR2111, miR169 and an miR827-like sequence with several miRNAs*, reporting a correlation between miRNA levels and the phosphorus (P) and/or nitrogen (N) status in the plant [54]. However, unlike the miR399/miR399* couple, only miR2111 is transported along with the phloem stream following a long-distance path, as its respective star sequence, miR2111*, seems to be less mobile [61]. Deeper insights into miR2111 mobility were recently provided in *Lotus japonicus* where, once produced in the leaf, the miRNA was observed to translocate to the root to modulate rhizobial infection through the post-transcriptional regulation of the symbiosis suppressor Too Much Love [62].

Experiments carried out in *Nicotiana benthamiana* demonstrated that miR172 is also translocated through the phloem into distal tissues following the source-to-sink direction [97], and it affects plant architecture and tuberisation in potatoes (*Solanum tuberosum*), in association with miR156 [55,57]. Evidence collected in potato thus suggests that miR172 is a key regulator of the tuberisation process, acting downstream of the tuberisation repressor photoreceptor phytochrome B (PHYB) factor and upstream of the tuberisation promoter homeodomain protein BEL5 [57]. Accordingly, transgenic potato lines overexpressing miR156 exhibit several morphological alterations and produce aerial tubers under inductive conditions. The demonstrated accumulation of miR156 in phloem vessels, and its consequent delivery to recipient tissues, play a pivotal role in controlling potato architecture and development [55] (Table 1).

Besides sncRNAs, long non-coding RNAs (lncRNAs), which are RNA molecules longer than 200 nucleotides without protein-coding potential, have acquired considerable importance in the last decade [98]. Accumulating evidence shows that plant lncRNAs are key regulators of gene expression and genome stability, and they also play relevant roles in the complex molecular networks regulating plant development, flowering and stress responses [99,100,101,102]. Other promising research avenues come from the fact that lncRNAs can function as a target mimic of miRNAs to regulate miRNA activity [103]. In this case, they act as competing endogenous RNAs because, being recognised by miRNAs via the target mimicry mechanism, they block the interaction between miRNAs and their targets. This is the case with the lncRNA IPS1 (Induced by Phosphate Starvation 1), which competes with PHO2 to bind miR399 [104]. Moreover, a high number of lncRNAs were isolated from the phloem of cucumber/watermelon grafts, indicating a potential long-distance trafficking of these molecules [103]. In the same study, the authors also pointed out that the abundance of lncRNAs, as well as their spread through the vasculature, is tissue-specific and influenced by P_i_ deficiency.

Taken together, this information provides compelling confirmation that the delivery of silencing effectors can be modulated by environmental cues, therefore adding a further level of complexity to the regulatory machinery associated with small RNA systemic silencing.

## 3. Spread of Small RNA-Directed DNA Methylation and Implications in Epigenetic Inheritance and Environmental Adaptation

Epigenetic modifications induced by mobile small RNA signalling represent another source for plant phenotypic variations, acting as a parallel system to genetic differences in influencing heritable variation, plant plasticity and, ultimately, environmental adaptation. Particularly, RdDM events associated with transcriptional gene silencing can take control of a wide array of biological processes, including genome stability, by preventing mobility of transposable elements (TE), development, imprinting, stress responses and stress memory [105].

Establishment of the ‘canonical’ RdDM cascade depends on the production of 24-nt siRNAs generated by DCL3 activity following synthesis of RNA precursors by RNA polymerase IV (Pol IV) and RDR2. Once formed, the 24-nt siRNAs are exported to the cytoplasm where they are loaded onto AGO proteins (most frequently AGO4). Subsequently, the AGO-siRNA module is imported back into the nucleus, where it targets chromatin-bound scaffold transcripts produced by Pol V at sites of methylation [35,106]. Although less frequently, ‘non-canonical’ RdDM pathways exist in plants and are probably activated to ensure the translational repression of active TEs that were originally targets of PTGS [107,108]. The ‘non-canonical’ RdDM scenario thus involves PTGS components, including Pol II and 21 to 22-nt sncRNAs, that serve as a guide for domains rearranged methyltransferases 2 (DRM2) to allow the synthesis of 24-nt siRNAs associated with RdDM establishment [35,107].

However, RdDM does not always rely on the accumulation of corresponding siRNAs, since alternative pathways, in which RdDM triggers are not siRNAs but long RNAs, do also exist, as recently revealed [109].

Moreover, it must be noted that the maintenance of RdDM can occur either in a small RNA-dependent or RNA-independent way. In the presence of an RNAi/RdDM trigger, DRM1 and DRM2 are the enzymes involved in de novo methylation of cytosines in CG, CHG and CHH contexts [110]. In the absence of an RNAi/RdDM trigger (namely RNA-independent maintenance of methylation), CHH methylation is lost, whereas methylation at CG and CHG contexts is maintained through the activity of MET1 [111] and CHROMOMETHYLASE3 (CMT3) [112] enzymes.

Another point deserving attention is the mobility of RdDM-based regulatory pathways. The first data in support of this theory was advanced by Molnar et al., who worked with grafted transgenic *Arabidopsis* plants [64]. Using triple dcl mutants lacking 22- to 24-nt siRNA biogenesis in either source or recipient tissue, these authors showed that transgene-derived, as well as endogenous, siRNAs were able to move across the graft junction via the phloematic stream, following the source-to-sink gradient and direct epigenetic modifications in the genome of receiving cells. Further results supporting this theory were achieved by Melnyk et al., who demonstrated that 24-nt siRNAs from the *Arabidopsis* shoot can reach the meristem cells in the root, where they guide the DNA methylation of three endogenous TEs [65]. Follow-up work by Lewsey et al. revealed that the same hc-siRNAs produced in the shoot can mediate the methylation of thousands of TE-associated loci in the root, further providing evidence of the role of the RdDM process in maintaining genome stability in distant cells [66].

Additionally, the movement of sncRNAs can mediate the transmission or re-establishment of DNA epigenetic marks in the next generation [43]. It was proposed that gene expression alterations are transmissible through meiotic or mitotic processes and can persist in newly generated cells, thus leading to transgenerational epigenetic silencing. Accordingly, results collected by diverse research groups indicated that siRNAs deriving from a mutated or silenced locus are delivered in the germ cells where they can either re-establish or maintain the epigenetic information [113,114]. This process is the basis of paramutations and TE silencing from one generation to another, and it directly depends on the capacity of the RdDM machinery to operate systemically and enter the germline.

Besides controlling the regulation of developmental patterning, epigenetic inheritance phenomena activated by small RNA trafficking have major implications in plant adaptability to changing environments [35]. In recent years, valuable experimental evidence supports that siRNA-dependent epigenetic modifications that serve as the basis of the reprogramming of stress-responsive gene expression can be transmitted across plant generations. It was thus hypothesised that these processes could lead to the occurrence of transgenerational epigenetic inheritance and eventually be associated with the establishment of stress memory in the offspring [43,115]. Investigation of the biology underlying stress memory has become an attractive research matter among plant physiologists. The existing knowledge on this topic indicates that RdDM processes can be alternatively connected with the gain or, more frequently, loss of heritable epigenetic variations, as was recently demonstrated by a number of studies conducted on the offspring of *Arabidopsis* plants previously exposed to pathogen infection [116,117]. A deeper insight into these subjects was later provided by experiments performed on in vitro regenerated rice plantlets grown in osmotic stress conditions [118]. According to this information, only the first of the two conditions relies on the production of sncRNAs, while the latter occurs when the RdDM-associated silencing pathway is turned off. Interestingly, it was proposed that, under high infection pressure, the RdDM machinery is inhibited in order to foster a priming response through the hypomethylation of defence genes. Conversely, in environments with low infection risk, defence/stress-responsive genes are silenced via the establishment of the RdDM pathway, thus reducing the fitness cost of disease resistance [119]. From an evolutionary perspective, the switch between establishment/amplification and loss of epigenetic marks in recipient/germline cells represents a clever strategy that allows for rapid plant adaptation to changing environments [43].

Additionally, changes in DNA methylation profiles can affect other facets of plant response to stress, including phenomena of plant environmental plasticity [120]. A profound understanding of these mechanisms is still far from being reached, and more research efforts must be made in this direction. However, available information indicates a contribution of sncRNA-directed epigenetic variations in influencing the transcriptome plasticity of plants in response to environmental constraints [121,122].

Although miRNA activity is commonly discussed considering the post-transcriptional regulation of genes, many of them have recently emerged as key epigenetic regulators that act as upstream triggers of the biogenesis of secondary siRNAs involved in RdDM pathways [123,124]. One of the most known miRNA-siRNA regulatory modules is the activation of tasiRNA biogenesis via miR173-, miR390- or miR828-guided cleavage of *TAS1*, *2*, *3* or *4* transcripts [8,12]. As in tasiRNAs, the synthesis of either 21- or 24-nt long-phasiRNAs, a class of still poorly characterised secondary siRNAs exclusively isolated in plants, directly depends on the slicing of *PHAS* transcripts, respectively promoted by miR2118 and miR2275 [37,125]. In parallel to the regulation of tasiRNAs and phasiRNAs, conserved miRNAs are also implicated in the biosynthesis of epigenetically activated siRNAs (easiRNAs) from transcriptionally reactivated TEs. Experimental evidence in support of this theory was provided by Slotkin et al. [126] and later by Creasey et al. [123], who showed that several miRNA sequences, including miR156, miR159, miR172 and miR390, are able to direct the generation of RDR6-dependent 21-nt easiRNAs from TE loci in *Arabidopsis* pollen cells during epigenetic reprogramming. This regulatory cascade exerts an important biological function as it protects the offspring from genome instability caused by the reactivation of TEs, a role particularly crucial in preventing transgenerational retro-transposition in plants subjected to stress [127]. Notably, most of the miRNAs involved in transcriptional silencing were often found in association with the control of plant stress-adaptive responses [128]. This is true in the case of miRNAs isolated in plants exposed to salinity stress, in which several stress-responsive genes undergo epigenetic modifications determined by specific miRNA regulation [115,129]. Progress in high-throughput sequencing technology has particularly allowed for the identification of many conserved miRNA targeting transcripts from stress-responsive genes, as well as tasiRNAs [18,24,130]. Interestingly, some of those miRNAs, such as the highly conserved miR156, are capable of movement within the plant [55] and have been shown to play important roles in the induction of stress memory phenomena [131].

## 4. Concluding Remarks

The biological functions of sncRNAs, and miRNAs in particular, have been widely explored in recent years. Particularly promising advances in this field were achieved using artificial miRNAs in functional genomics studies [132] and the application of CRISP/Cas9 technology-based approaches for the cloning of miRNA genes [133].

Nevertheless, more investigations are needed to elucidate the role and the activation of these molecules in response to environmental cues, particularly in multiple stress conditions. In fact, it has recently begun to emerge that a variety of environmental alterations can differently affect miRNA production and stability by directly altering the main enzymes related to their entire biogenesis pathways. In response to specific stress signals, the whole machinery associated with miRNA production can be turned down through the establishment of feedback regulatory mechanisms [44]. An excellent example is represented by 22-nt miR168 molecules that are produced instead of the 21-nt long isoforms of the same miRNA and that are preferentially complexed with AGO10 to trigger the synthesis of secondary siRNAs from AGO1 loci [134].

All the investigations discussed in the present review highlight the high level of complexity related to the establishment and amplification of RdDM and PTGS signalling pathways. They certainly emphasise the importance of studying small RNA-mediated regulation in parallel with the effects of sncRNA mobility on a wide range of physiological processes, from developmental patterning to stress signalling cascades, to epigenetic inheritance. Particularly, more efforts should be made to unveil the targets of miRNAs with considerations that, unlike miRNAs, target transcripts are often diverse amongst different plant species. In fact, although studies on stress-responsive miRNAs have increased significantly for several plant species, physiological responses mediated by miRNAs in the presence of environmental stresses must be further explored. From a future perspective, this is also very important considering the potential use of miRNAs as key targets for improving stress resistance in plants [3,130]. The same is true for gaining more insights into the biological role of mobile sncRNAs as systemic silencing effectors in crops other than model species.

A deeper understanding of this information would be very crucial to opening novel and interesting avenues for breeding applications, including the development of stress-resilient crops that better respond to ongoing climate change.

## Figures and Tables

**Table 1:** Summary of mobile endogenous small RNAs identified in plants so far. PD: plasmodesmata; OE: over-expressing; TE: transposable elements; RT-qPCR: real-time quantitative polymerase chain reaction.

Small RNA Type	Size (nt)	Biological Process	Transport Route	Plant Species	Experimental Strategy	References
miR390	21	Leaf polarity	cell-to-cell through PD	*Arabidopsis*	in situ hybridization	[50]
miR165/166	21	Root development and cell differentiation	cell-to-cell through PD	*Arabidopsis*	in situ hybridization, RT-q PCR, mutants, microarrays, histological assays	[51,52]
miR394	20	Shoot apical meristem formation	cell-to-cell through PD	*Arabidopsis*	mutant screen	[53]
miR169	20 to 21	Nitrogen and phosphate limitation	shoot-to-root	*Arabidopsis*, rapeseed	phloem sap analysis, high throughput sequencing, stem loop RTqPCR	[54]
miR156	21	Regulation of plant architecture and tuberization	shoot-to-root	potato	phloem sap analysis, grafted plants, stem loop RTqPCR, miR156-OE lines	[55]
miR172	21	Tuberization process, salt stress tolerance	root-to-shoot	potato, soybean	grafting experiments, miR172-OE plants, in situ hybridization	[56,57]
miR399	20 to 21	Phosphate homeostasis	shoot-to-root	*Arabidopsis*, rapeseed, pumpkin, tobacco	grafting experiments, sequencing of phloem exudate, stem loop RTqPCR, Northern blot, mutants	[58,59,60]
miR399*	21	Response to phosphate starvation and nitrogen availability	shoot-to-root	*Arabidopsis*, rapeseed	grafting experiments, sequencing of phloem exudate, stem loop RTqPCR	[54]
miR398	21	Response to copper deprivation	shoot-to-root	*Arabidopsis*, rapeseed	grafting experiments, sequencing/Northern blot of phloem exudate, stem loop RTqPCR	[54,58]
miR395	21	Sulphate homeostasis	shoot-to-root	rapeseed	grafting experiments, Northern blot/sequencing of phloem sap samples	[58]
miR827		Phosphate starvation	shoot-to-root	*Arabidopsis*, rapeseed	grafting experiments, sequencing of phloem sap samples, stem loop RTqPCR	[54,61]
miR2111	21	Phosphate starvation, rhizobial infection	shoot-to-root	*Arabidopsis*, rapeseed, *Lotus japonicus*	sequencing of phloem exudate, grafting experiments, stem loop RTqPCR	[54,61,62]
*TAS3*-derived secondary siRNAs (tasiR-ARFs)	21	Establishment of the adaxial–abaxial leaf polarity, developmental patterning	cell-to-cell	*Arabidopsis*	in situ hybridization	[50]
TE-derived siRNAs	21–24	TE methylation and maintenance of genome stability during reproduction	pollen vegetative cell to sperm cells	*Arabidopsis*	transgenic plants, microarray, high-throughput sequencing	[63]
hc-siRNAs	22–24	DNA methylation in CHH contexts (TEs) in root meristem	shoot-to-root	*Arabidopsis*	grafting, transgenic plants, high-throughput sequencing	[64,65,66]
